# Impact of stair climbing volume on patellofemoral cartilage: a dose-response analysis from the osteoarthritis initiative reveals elevated risk at middle levels

**DOI:** 10.3389/fmed.2025.1699297

**Published:** 2025-11-24

**Authors:** Yaohui Yang, Zhiyao Zhao, Hairui Zhang, Fangzheng Zhou, Xiaoning Liu

**Affiliations:** Orthopaedic Medical Center, Second Hospital of Jilin University, Changchun, China

**Keywords:** patellofemoral joint, osteoarthritis, cartilage, stair climbing, the osteoarthritis initiative

## Abstract

**Objective:**

To evaluate the impact of stair climbing volume on cartilage degeneration in the patellofemoral joint, with a particular focus on the medial/lateral patellar and trochlear regions.

**Methods:**

Utilizing data from the osteoarthritis initiative (OAI) cohort, we analyzed 581 participants with baseline and 24-month follow-up MRI assessments. Cartilage damage was evaluated using the MRI Osteoarthritis Knee Score (MOAKS), with subregional stratification (medial/lateral patella, medial/lateral trochlea). Stair-climbing volume was categorized as low (0–4 flights/week), middle (5–6 flights/week), or high (> 6 flights/week). Logistic regression models adjusted for BMI, age, and sex assessed the dose-response relationship between stair climbing and cartilage deterioration across patellofemoral subregions.

**Results:**

A total of 581 participants were included (59.4% female, age 61.6 ± 8.9 years, BMI = 30.7 ± 4.7 kg/m^2^); 13.6, 6.4, and 80% of the participants reported stair climbing of 0–4, 5–6, and > 6 flights per week, respectively. Middle-volume stair climbing was associated with an increased risk of worsening MOAKS cartilage score [adjusted OR (95% CI): 3.068 (1.230–7.652)]. Stratified analysis showed that middle-volume stair climbing was associated with worsening MOAKS cartilage score of trochlear surface cartilage [adjusted OR (95% CI): 4.495 (1.148–17.592)].

**Conclusion:**

Middle-volume stair climbing was associated with greater progression of patellofemoral joint cartilage deterioration, particularly in the trochlear region, suggesting that mechanical loading patterns during stair climbing may influence subregional cartilage vulnerability.

## Introduction

Patellofemoral arthritis is a chronic degenerative disease characterized by the destruction of articular cartilage and the formation of osteophytes (1). It is a significant contributor to knee pain and functional impairment, with the incidence rate among adults reaching approximately 11–25% (2–4). The role of physical activity in the incidence and progression of patellofemoral arthritis remains an active area of investigation. Stair climbing is a prevalent form of physical activity that effectively enhances lower limb strength and improves cardiopulmonary function. Importantly, stair climbing occurs not only as a structured exercise but also incidentally in daily lifef physas at home, in the workplace, or within certain occupationshat effectively enhances lower limb strength and improves cardiopulmonary functi (5, 6). However, stair climbing generates increased patellofemoral contact forces. While such loading is a normal physiological occurrence, repetitive or excessive loads that exceed an individual an individualpotolerance may play a role in osteoarthritis pathophysiology by altering cartilage mechanical properties and promoting local inflammatory or catabolic responses (7–9).

Several studies have reported that activities involving prolonged squatting, climbing, and biomechanical loading during sustained knee flexion postures demonstrate a significant correlation with elevated risk of patellofemoral arthritis, particularly in occupations requiring repetitive kneeling maneuvers (10–16). These movements place significant mechanical stress on the patellofemoral joint, potentially leading to cartilage degeneration over time (12). However, no studies have reported the relationship between stair climbing volume and patellofemoral arthritis. The osteoarthritis initiative (OAI) cohort offers an opportunity to evaluate the impact of stair climbing volume on the progression of cartilage damage associated with patellofemoral arthritis.

This study aimed to explore the association between stair climbing volume and patellofemoral arthritis-related cartilage damage. We hypothesized that middle-volume stair climbing per week would significantly increase patellofemoral articular cartilage damage.

## Materials and methods

### The osteoarthritis initiative

The osteoarthritis initiative (OAI), a longitudinal multicenter observational research program, is administered under the auspices of the National Institutes of Health (NIH), an agency within the U.S. Department of Health and Human Services (HHS). The OAI study enrolled 4,796 participants aged 45–79 from 2004 to 2006, including symptomatic knee osteoarthritis (OA) patients as well as those at high risk (17). The OAI research team comprises specialists from various research centers, such as the University of Maryland School of Medicine, Ohio State University, University of Pittsburgh, Rhode Island Memorial Hospital, and the University of California, San Francisco. Via this research project, OAI strives to offer a complete database illustrating the disease’s natural course and imaging and biochemical biomarker data. This study was approved by the Institutional Review Boards at each participating OAI site and Baylor College of Medicine (approval number: 10-00532). All public data can be accessed at https://nda.nih.gov/oai/.

### Magnetic resonance imaging pulse sequence protocols

Four identical Siemens 3T MRI scanners were used to acquire MRI images of both knees of all OAI participants during each follow-up visit. The MRI pulse sequence protocol comprised a coronal two-dimensional (2D) intermediate-weighted (IW) turbo spin-echo, sagittal three-dimensional (3D) dual-echo at steady-state (DESS), and coronal and axial multiplanar reformations of the 3D DESS. It also included sagittal IW fat-saturated (FS) TSE sequences (17, 18).

### Semi-quantitative radiographic evaluation of patellofemoral joint

The MRI Osteoarthritis Knee Scoring (MOAKS) is a validated semi-quantitative MRI patellofemoral osteoarthritis scoring method that was independently performed by 2 experienced musculoskeletal radiologists on knee MRI of 600 participants. MOAKS scores the surface and full-thickness of the medial patella, lateral patella, medial anterior femur (medial trochlea), and lateral anterior femur (lateral trochlea) according to the percentage of the subregion affected by the lesion. The size of any cartilage lesions is scored on a 4-point scale (0: none, 1: < 10% of the surface or full-thickness area of the region, 2: 10–75% of the surface or full-thickness area of the region, 3: > 75% of the surface or full-thickness area of the region). Patellofemoral cartilage worsening was reported if the cartilage score increased during the interval (baseline to 24-month follow-up intervals) and was categorized as surface, thickness, or any cartilage deterioration. During the follow-up evaluation, a threshold of 0.5 was defined to demarcate that although clinical scores remained unchanged from baseline, marked clinical deterioration had manifested, which was called within-grade worsening and was also considered a worsening (19, 20). The progress of cartilage scores of the patella and trochlea were compared and analyzed respectively, and the main site of injury was analyzed. Also, considering that participants may have undergone total knee arthroplasty (TKA) between baseline and 24-month follow-up intervals, participants who underwent TKA in any knee were supposed to have patellofemoral cartilage deterioration.

**FIGURE 1:**
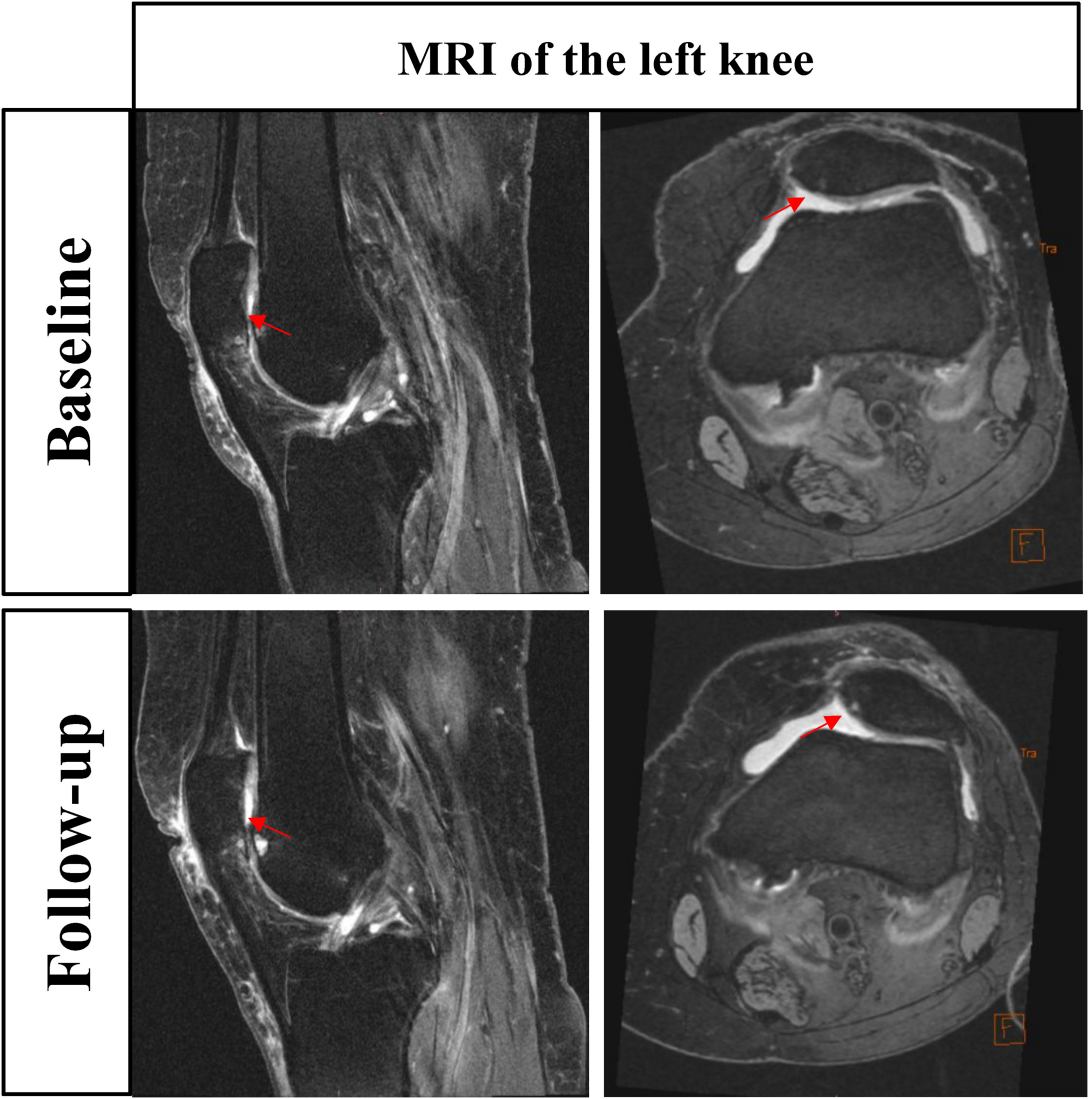
MRI images depicting patellofemoral cartilage changes in the knee of a 72-year-old participant engaged in middle-volume stair climbing after 2 years.

For example, a 72-year-old Caucasian female participant with a BMI of 26.1 kg/m^2^ engaged in middle-volume stair climbing activity. The left knee MRI, specifically the intermediate-weighted fat-saturated TSE sequence, is shown in Figure 1. At baseline, the sagittal (A) and axial (B) MRI of the left knee revealed chondral damage in the patellofemoral joint, with the lateral patellar cartilage scoring 2 on the semiquantitative MRI Osteoarthritis Knee Score (MOAKS) for both surface and full-thickness involvement (affecting 10–75% of the cartilage surface and thickness). At the 24-month follow-up, the sagittal (C) and axial (D) MRI showed deterioration of lateral patellar cartilage damage, with MOAKS scores of 3 for surface involvement (> 75% area) and 2 for thickness involvement (10–75% thickness). Red arrows indicate the sites of patellofemoral cartilage damage.

### Evaluation of stair climbing

During the baseline assessment protocol, participants underwent structured data collection procedures involving stair climbing. A validated questionnaire administered through interviewer-assisted recall specifically inquired: “How many flights of stairs have you climbed up in the past 7 days (one flight equals 10 steps)?” Participants reported the number of stairs climbed. Self-documented ascent frequencies were subsequently categorized through standardized stratification protocols utilizing the OAI dataset. The participants are divided into three groups: (A) low-volume stair climbing exercise, defined as 0–4 flights per week; (B) middle-volume stair climbing exercise, defined as 5–6 flights per week; and (C) high-volume stair climbing exercise, defined as more than 6 flights per week.

### Eligibility criteria

**FIGURE 2:**
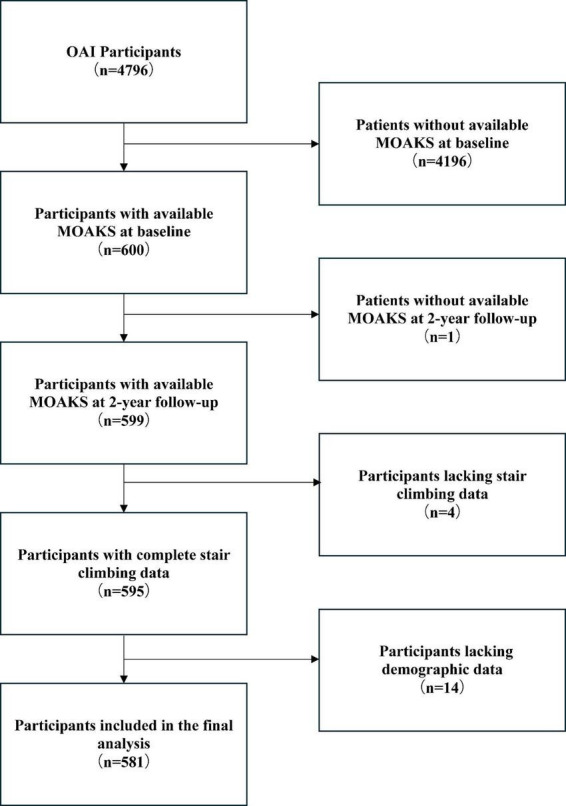
Flow diagram reflecting how 581 participants were selected from the original 4,796 osteoarthritis initiative participants. OAI, osteoarthritis initiative; MRI, magnetic resonance imaging; MOAKS, MRI Osteoarthritis Knee Scoring.

Participants were selected according to the following criteria. The osteoarthritis initiative (OAI) baseline cohort comprised 4,796 enrolled participants at study initiation. From this cohort, 600 (12.5%) participants were selected based on the MOAKS. Application of exclusion criteria led to the removal of one (0.2%) participant due to missing 24-month follow-up data, four (0.7%) due to incomplete baseline stair climbing data, and fourteen (2.3%) due to the absence of baseline demographic information. Following comprehensive eligibility screening, 581 (96.8% of the initially selected 600) qualified participants formed the final analytical cohort, with detailed selection procedures diagrammatically presented in Figure 2.

### Statistical analysis

The demographic characteristics [age, sex, body mass index (BMI)], volume of stair climbing exercise, and MOAKS assessments, with quantitative analysis conducted at initial baseline evaluation and 24-month follow-up intervals. Stair climbing volume, the primary exposure variable, was categorized into three levels: low-volume (0–4 flights per week), middle-volume (5–6 flights per week), and high-volume (> 6 flights per week). The primary outcome was patellofemoral cartilage deterioration within 24 months, defined as an increase in the cartilage damage subscore of the MOAKS. Model 1 was an unadjusted model that evaluated the crude association. Model 2 was further adjusted for potential confounders (age, sex, and BMI), as these variables are known to influence knee cartilage metabolism and loading patterns. To further explore regional differences, the patellofemoral cartilage was divided into two anatomical subregions—the patella and the trochlea—and the ORs and 95% CIs for cartilage deterioration were calculated separately for each subregion. Model 3 performed a sensitivity analysis by incorporating baseline cartilage damage into the analysis. Statistical comparisons between subgroups were performed using ANOVA for continuous variables and Pearson’s chi-square test for categorical variables. Logistic regression modeling quantified dose-dependent relationships between stair climbing volume and the worsening of patellofemoral cartilage. The Hosmer-Lemeshow test was used to evaluate the goodness of fit of the logistic regression model. Obesity (high BMI) is considered a potential risk factor for patellar cartilage degeneration (21). The selection of these covariates was based on the following rationale: age and sex are key determinants of cartilage composition and osteoarthritis risk, while BMI reflects obesity-related mechanical loading and metabolic effects that may influence patellofemoral cartilage integrity. The adjusted models were subsequently analyzed after adjusting for potential confounders such as BMI, age, and sex. In addition, we conducted a sensitivity analysis by including baseline cartilage damage status as an additional covariate in Model 3 to assess whether pre-existing cartilage lesions influenced the association between stair climbing volume and cartilage degeneration. All statistical analyses were performed using DSS Statistics 25, and statistical significance was defined as *P* < 0.05 in the analytical process.

## Results

The study cohort (*n* = 581) exhibited a mean age of 61.6 ± 8.9 years and BMI of 30.7 ± 4.7 kg/m^2^, with female predominance (59.4%) in baseline demographic characteristics. Among 581 participants, 79 (13.6%), 37 (6.4%), and 465 (80.0%) participants reported climbing < 4 flights, 5–6 flights, and more than 6 flights of stairs in a week, respectively. Baseline demographic profiles are presented in Table 1. ANOVA results indicated a significantly higher proportion of females engaged in low-volume stair climbing (*p* = 0.024). Pearson’s chi-square test showed that participants involved in high-volume stair climbing had a lower BMI (*p* = 0.013). Our analyses revealed no statistically significant difference in age across the study participants (Table 1).

**TABLE 1:** Summary of group characteristics.^a^

Variable	Total (*n* = 581)	Low-volume (*n* = 79)	Middle-volume (*n* = 37)	High-volume (*n* = 465)	*P*
Age, y	61.6 ± 8.9	63.1 ± 8.8	60.5 ± 9.0	61.5 ± 8.9	0.242^b^
Sex					0.024^c^
Male	236(40.6%)	24(30.4%)	21(56.8%)	191(41.1%)	
Female	345(59.4%)	55(69.6%)	16(43.2%)	274(58.9%)	
Race					0.059^c^
White	461(79.3%)	54(68.4%)	29(78.4%)	378(81.3%)	
Black	104(17.9%)	23(29.1%)	6(16.2%)	75(16.1%)	
Other	16(2.8%)	2(2.5%)	2(5.4%)	12(2.6%)	
BMI, kg/m^2^	30.7 ± 4.7	32.0 ± 5.5	31.5 ± 4.8	30.4 ± 4.6	0.013^b^

*^a^*Values are presented as mean ± SD or n (%).

*^b^*Analysis of variance.

*^c^*Pearson chi-square test. BMI, Body Mass Index.

We used participants with low-volume stair climbing exercises as the reference group. Longitudinal analysis revealed a significant dose-response relationship between incremental stair-climbing exertion levels and progressive patellofemoral cartilage degradation over the 24-month observational period (Table 2). In model 1, the Odds Ratio (OR) of surface cartilage progression in participants with middle-volume stair climbing was 2.853 (95% CI: 1.000–8.136), and the OR of any cartilage progression was 2.515 (95% CI: 1.035–6.114), which were statistically significant. After adjusting for covariates such as BMI, sex, and age in model 2, the results indicated that the OR for surface cartilage progression in participants with middle-volume stair climbing was 3.217 (95% CI: 1.105–9.368), and the OR for any cartilage progression was 3.068 (95% CI: 1.230–7.652), both remaining statistically significant. Additionally, while the OR was greater than 1 for patellofemoral thickness in participants with middle-volume stair climbing and for cartilage in those with high-volume stair climbing, suggesting potential cartilage deterioration, these results were not statistically significant. In the sensitivity analysis adjusted for baseline cartilage damage (Model 3), middle-volume stair climbing remained significantly associated with patellofemoral cartilage deterioration (adjusted OR = 3.234, 95% CI: 1.111–9.417, *p* < 0.05), indicating that baseline lesions did not substantially confound the observed association (Supplementary Table 1).

**TABLE 2:** The MOAKS progression of patellofemoral joint cartilage over 24 months among participants with varying intensities of stair climbing.

	Surface (medial/ lateral)	Thickness (medial/ lateral)	Any
Model 1	OR (95% CI), *p*-value	Referent	Referent
Low-volume	Referent		
Middle-volume	2.853 (1.000–8.136), **0.050**	1.303 (0.467–3.637), 0.614	2.515 (1.035–6.114), **0.042**
High-volume	1.340 (0.615–2.920), 0.461	1.377 (0.715–2.652), 0.339	1.473 (0.796–2.726), 0.217
Model 2	OR (95% CI), *p*-value		
Low-volume	Referent	Referent	Referent
Middle-volume	3.217 (1.105–9.368), **0.032**	1.537 (0.539–4.382), 0.421	3.068 (1.230–7.652), **0.016**
High-volume	1.504 (0.680–3.328), 0.314	1.626 (0.829–3.189), 0.157	1.754 (0.930–3.307), 0.083

Bold values indicate statistically significant results (*p* < 0.05). OR, odds ratio; CI, confidence interval. Model 2: adjusted for age, BMI and sex.

Further analysis that subdivided the patellofemoral cartilage into patella and trochlear components showed that trochlear surface cartilage injury in the middle-volume stair climbing group was statistically significant compared to the reference group (Model 2 OR of 4.495 (95% CI: 1.148–17.592), *p* = 0.031) (Table 3).

**TABLE 3:** The MOAKS progression of patellar or trochlear cartilage over 24 months among participants with varying intensities of stair climbing.

	Surface (medial/ lateral)	Thickness (medial/ lateral)	Any
Patella model 1	OR (95% CI), *p*-value	Referent	Referent
Low-volume	Referent		
Middle-volume	1.794 (0.453–7.112), 0.406	1.505 (0.493–4.596), 0.473	1.903 (0.682–5.310), 0.219
High-volume	1.431 (0.548–3.740), 0.465	1.355 (0.647–2.839), 0.421	1.543 (0.764–3.119), 0.227
Patella model 2	OR (95% CI), *p*-value	Referent	Referent
Low-volume	Referent		
Middle-volume	1.985 (0.489–8.057), 0.338	1.889 (0.604–5.912), 0.274	2.343 (0.819–6.697), 0.112
High-volume	1.592 (0.597–4.244), 0.353	1.656 (0.775–3.542), 0.193	1.843 (0.895–3.797), 0.097
Trochlear model 1	OR (95% CI), *p*-value	Referent	Referent
Low-Volume	Referent		
Middle-volume	3.629 (0.957–13.757), 0.058	0.411 (0.046–3.650), 0.425	2.355 (0.704–7.874), 0.164
High-volume	1.065 (0.361–3.148), 0.909	1.205 (0.457–3.175), 0.706	1.240 (0.509–3.018), 0.636
Trochlear model 2	OR (95% CI), *p*-value	Referent	Referent
Low-volume	Referent		
Middle-volume	4.495 (1.148–17.592), **0.031**	0.433 (0.048–3.897), 0.455	2.697 (0.787–9.238), 0.114
High-volume	1.271 (0.422–3.833), 0.670	1.328 (0.495–3.567), 0.573	1.435 (0.579–3.588), 0.436

Bold values indicate statistically significant results (*p* < 0.05). OR, odds ratio; CI, confidence interval. Model 2: adjusted for age, BMI and sex.

## Discussion

In this cohort study involving 581 participants, we found that middle-volume stair climbing was significantly associated with patellofemoral cartilage lesions, whereas high-volume stair climbing did not show a statistically significant association. The participants of this study were recruited from the community and are more representative of the general population.

Previous studies have shown that physical activity is associated with the risk of patellofemoral arthritis (10–16). Haj-Mirzaian et al. (10) showed that frequent kneeling activities were associated with an increased risk of patellofemoral arthritis (OR, 2.33 95%CI: 1.08–5.06). Another study showed that frequent flexion activities increased the risk of patellofemoral cartilage impairment (OR, 3.63 95%CI 1.22–7.79) (13). However, these studies have mainly focused on occupational or repetitive loading, and no research has yet linked stair climbingellofemoral arthritisicipants with middleion and osteoarthto patellofemoral cartilage degeneration. Using longitudinal MRI data, the present study is the first to reveal that even middle-volume stair climbing is significantly associated with patellofemoral cartilage deterioration, particularly in the trochlear region, thereby extending the risk of early degeneration to everyday life scenarios. Climbing stairs is a form of daily life activity for modern people and is inevitably involved every day. Therefore, we chose people who climb stairs at a low volume as the control group to avoid the potential bias caused by selecting people who do not exercise at all as the control group. This allows us to more accurately assess the risk of patellofemoral joint injury caused by the volume of stair climbing, making the research results more comprehensive and objective, and able to comprehensively reflect the knee injury situation of stair climbing at different volumes.

Potential biomechanical mechanisms may explain the relationship between stair climbing volume and worsening patellofemoral cartilage damage. First, stair climbing increases the contact mechanics of the patellofemoral joint, and the pressure on the patellofemoral joint when the knee is flexed to 30 degrees is twice that of walking on the ground (8). Second, increased mechanical loading may increase cartilage damage and promote increased expression of proinflammatory cells (22). A plausible explanation for the lower risk observed in the high-volume group is that long-term high-volume stair climbing may enhance muscle strength and joint stability, thereby providing better support to the patellofemoral joint and mitigating cartilage degeneration caused by repetitive loading. Consistent with previous studies, we found that middle-volume stair climbing exercise was associated with patellofemoral arthritis.

However, this cohort study has some limitations. First, there was a significant difference in gender between the groups, with a higher proportion of females in the low-volume stair climbing group and a higher proportion of males in the high-volume group (*p* < 0.05). Although this difference was controlled in model 2, this bias may limit the general applicability of the results and should be interpreted in conjunction with the included patients. Secondly, the sample size and follow-up time of the study may be insufficient, and further exploration is needed in combination with a broader database. Third, the baseline quantification of stair climbing volume levels introduces potential longitudinal participation bias, given the temporal variability inherent in physical activity patterns across the observational period. To address this limitation, we extracted stair climbing volume at 24-month follow-up intervals, and compared it with stair climbing volume at baseline, and these data showed no significant difference. Fourth, since stair-climbing volume was self-reported, recall bias may exist. In particular, participants in the high-volume group may have overestimated their activity levels, which could explain the non-significant association observed. Future studies could employ objective measurement tools, such as accelerometers, to improve the accuracy of activity classification. Finally, the number of participants in each group was relatively small, which may have limited the statistical power of our analysis. This limited sample size likely contributed to the wide confidence intervals observed for some odds ratios, indicating a degree of imprecision in the estimated effect sizes. Therefore, these results should be interpreted with caution and confirmed in larger, prospective studies.

As an observational study, our findings demonstrate correlation, not causation. Although we adjusted for key confounders such as age and BMI, the possibility of residual confounding cannot be excluded. Factors such as occupational knee loading, participation in other forms of physical activity, and lower-limb alignment were not fully assessed and may have influenced the observed associations. Therefore, the results should be interpreted as indicative of potential relationships rather than definitive causal effects.

Schiphof et al. found that crepitus in the knee is characterized as a clear, palpable vibratory friction sound, and crepitus is an early indicator of patellofemoral arthritis (23). Crossley and Peat et al. discovered that pain in the patellofemoral joint may be a clinical manifestation of moderate to severe patellofemoral arthritis (24, 25). Utting et al. identified that anterior knee pain may be one of the contributing factors to patellofemoral arthritis (26). Zhao et al. found that the sulcus angle and patellofemoral index are significantly associated with patellofemoral arthritis from a statistical standpoint (27). Waiteman et al. observed that female patients with patellofemoral pain and knee crepitus exhibit a reduced knee flexion angle when climbing stairs (28). Future research could focus on the relationship between the above clinical manifestations, knee function, positive findings from physical examinations such as the patellar grind test, and the volume of stair climbing. As a simple and functional indicator of daily activity, the total amount of stair climbing can help clinicians assess patientsove clinical manifestations, knee function, positive findings terns across -intensity activities due to knee pain. Although the total amount alone is insufficient for diagnostic purposes, it can complement other clinical measures to evaluate functional status or guide rehabilitation strategies. When conducting such studies, it is important to minimize the influence of patient subjectivity on the results to enhance the credibility of the findings. A comprehensive and integrated analysis of the relationship between stair climbing volume, patellofemoral arthritis, and knee cartilage changes should be conducted using symptomatology and imaging techniques.

Patellofemoral arthritis and tibiofemoral arthritis are both common manifestations of knee osteoarthritis, and they may also coexist (2, 3, 29). This study found that the relationship between stair climbing volume and patellofemoral cartilage deterioration may be moderated by individual risk factors. Individuals with better muscle strength may not necessarily experience damage even with high-volume stair climbing and may, in fact, benefit from improved joint stability. Therefore, public health messages should emphasize individualized risk assessment rather than broadly advising older adults to reduce stair climbing. This study focuses solely on patellofemoral arthritis. Future research could further investigate the relationship between stair climbing volume and tibiofemoral arthritis, and the combined effects when both conditions coexist. This would contribute to a more comprehensive understanding of the link between knee osteoarthritis and fundamental lifestyle factors and provide more precise guidance for clinical interventions.

## Conclusion

Our results showed that the middle-volume stair climbing group had significantly increased patellofemoral cartilage damage after 2 years of follow-up compared with the low-volume stair climbing control group. In clinical recommendations, factors such as BMI and overall joint health should be comprehensively considered. Middle-volume stair climbing may pose risks only for certain high-risk individuals, whereas high-volume stair climbing may be safe and even beneficial for others. Future public health guidelines should promote personalized assessment and intervention rather than universally recommending reduced stair climbing volume among older adults.

## Data Availability

The datasets presented in this study can be found in online repositories. The names of the repository/repositories and accession number(s) can be found at: The datasets generated during the current study are publicly available and accessible through the OAI https://nda.nih.gov/oai.
