# Supplementary material for: Impact of stair climbing volume on patellofemoral cartilage: a dose-response analysis from the osteoarthritis initiative reveals elevated risk at middle levels
Source: Front Med (Lausanne). 2025 Nov 24;12:1699297. doi: 10.3389/fmed.2025.1699297 (PMC12682809; doi:10.3389/fmed.2025.1699297)
Supplement: Supplementary file 1 [file Table_1.docx]

Supplementary Table 1 The MOAKS progression of patellofemoral joint cartilage over 24 months among participants with varying intensities of stair climbing, adjusted for baseline cartilage status

|  | Surface(medial/lateral) | Thickness(medial/lateral) | Any |
| --- | --- | --- | --- |
| Model 3 | OR (95% CI), p value |  |  |
| Low-Intensity | Referent | Referent | Referent |
| Middle-Intensity | 3.234 (1.111-9.417), **0.031** | 1.539 (0.540-4.386), 0.420 | 3.076 (1.233-7.672), **0.016** |
| High-Intensity | 1.490 (0.674-3.296), 0.325 | 1.622 (0.827-3.182), 0.159 | 1.745 (0.926-3.291), 0.085 |

OR, odds ratio; CI, confidence interval. Model 2: adjusted for age、BMI、sex and status of cartilage damage at baseline. Bold values indicate statistically significant results.
